# Use of glycosylated haemoglobin as diagnostic tool in Greenland: prevalence of diagnosed diabetes mellitus

**DOI:** 10.1186/1758-5996-5-59

**Published:** 2013-10-09

**Authors:** Line Damsgaard, Michael Lynge Pedersen

**Affiliations:** 1 Queen Ingrid Health Care Center, Nuuk, Greenland; 2 Greenland Center for Health Research, University of Greenland, Nuuk, Greenland

**Keywords:** Diabetes mellitus, Greenlanders, Inuit, Haemoblobin A1C, Glycohemoglobin

## Abstract

**Background:**

The prevalence of undiagnosed diabetes mellitus (DM) in Greenland has been reported very high with only 30% of cases diagnosed. In 2010, glycosylated hemoglobin (A1C) was introduced as a diagnostic tool in Greenland. However, the current use of A1c is unknown as well as the current prevalence of diagnosed DM.

The aim of this study was firstly to estimate the use of A1C as diagnostic tool within the first 27 months after introducing the method and secondly to estimate the age and gender specific prevalence of diagnosed DM in Greenland in 2012.

**Methods:**

This study was perfomed as a cross-sectional register study using data from electronic medical records (EMR). To analyse the use of A1C as diagnostic tool:

A sample amongst all Greenlanders at or above age 35 old was used to determine the number of individuals screened with A1C within a 27 month period, excluding those already known to have DM.

To estimate the prevalence of diagnosted DM: Patients with DM were identified electronically using a statistic module run on data in the EMR. Age and gender specific prevalence was estimated using the Greenlandic population as of 1 January 2012 as the background population.

**Results:**

The test sample resulted in a study group of 1008 individuals from which 2.3% (23) were excluded because they were already known to have DM. Among the remaining 985, 13.6% were tested with A1C at least once during the 27 months of observation. DM was diagnosed in 7.5% (10) of the tested persons and in 1.0% of the whole group.

Regarding prevalence, a total of 920 patients with diagnosed DM were identified. The total prevalence among adults aged 20–79 years old was 2.20% (95% CI: 2.05-2.34) with no significant difference between genders.

**Conclusion:**

Testing for DM using A1C as diagnostic tool is used in Greenland. The prevalence of diagnosed DM in Greenland remains low although increasing. Undiagnosed DM may still be an important issue in Greenland.

## Introduction

Greenland has undergone a rapid transition during the last half century from a traditional Inuit society dominated by small communities, villages and settlements to a modern society [1]. Within a few decades (1950s to 1970s), Greenland was transformed from a traditional hunting society to a modern society where most people now rely on wage earning [1]. These profound social and cultural change have been followed by a health transition with an increasing prevalence of lifestyle related diseases like overweight, obesity, diabetes and ischemic heart diseases [2-7] similar to what has occured among Inuit in Alaska and Canada [8-10].

Thus, diabetes mellitus (DM) used to be very rare (less than 1% of the population) in Greenland [11]. However, epidemiological studies have indicated a high prevalence of diabetes at around ten percent among adult Greenlanders based on oral glucose tolerance tests [6] comparable to levels among Inuit and Native Indian populations in Canada and Alaska [12-15]. However, seventy to eighty percent of cases were undiagnosed indicating low diagnostic activity [6,16].

In 2008, a national diabetes program was implemented in the primary health care system in Greenland aiming to improve the diabetes care including diagnostic activity and screening for diabetic complications. Along with increasing quality of care, the prevalence of diagnosed type 2 diabetes mellitus (T2DM) rose by 19% within two years from 1.97% to 2.3% among adult Greenlanders at or above 40 years old [17]. However, the prevalence of diagnosed T2DM was still much lower than reported in the surveys of the population [6,16].

In order to optimize the diagnostic procedure of diabetes and to make case finding more feasible in Greenland, the diagnostic strategy was changed by the first of June 2010. From that date glycosylated hemoglobin (A1C) was introduced as a screening tool [18,19] in Greenland consequently replacing the former use of fasting whole blood glucose testing [20]. Neither the diagnostic activity nor the prevalence of diagnosed diabetes mellitus has been evaluated after the introduction of A1C as a diagnostic tool.

The aim of this study was first to describe within the first twenty-seven months after introducing A1C as a diagnostic strategy the diagnostic activity with A1C as a screening tool and second, to estimate the age and gender specific prevalence of diagnosed diabetes mellitus in Greenland in 2012.

## Materials and methods

The study was designed as two cross-sectional observational register studies of the Greenlandic population. Observational data were collected retrospectively through the Electronic Medical Record (EMR) implemented throughout the whole Greenlandic primary health care system in the autumn of 2007.

### Setting

Greenland is the largest island in the world covering an area of over 2 million km^2^[21]. The country is sparsely populated with approximately 56,000 inhabitants living along the coast in 18 towns and around 60 minor settlements. Furthermore, the country is divided into 4 governmental regions and 5 health regions [22]. Approximately 90% of the population is of Greenlandic origin (native Inuit); the last 10% are immigrants (mostly Danes) [21].

Primary health care is free to all inhabitants and provided locally in primary health care clinics. Since 2008 all patients diagnosed with diabetes mellitus have been coded with “D” in the EMR.

Measurements of A1C were performed at The Central Laboratory at Queen Ingrid Hospital in Nuuk, using Architecht® 8000T (Abbott Laboratories) based on an enzymatic technique. The Central Laboratory is a member of the Danish quality control system for laboratories, DEKS. In addition some of the analyses of A1C have been performed locally using DCA vantage® (Siemens Healthcare Diagnostics) based on an immunological technique. Results of the tests were recorded in the electronic lab card for Greenland (BBC) and/or directly in the lab card in the EMR. A confirmed value of A1C at or above 48 mmol/mol (6.5%) is used as diagnostic for diabetes mellitus in Greenland as recommended by the American Diabetes Association [18].

### Study populations and analysis

#### Use of A1C as diagnostic tool – study sample 1

The first study population was defined as anyone affiliated with a primary health clinic in Greenland, who was born on the 1st of any month of the year before first of January 1978. Thus only adults at or above 35 years old where included.The cut-off age was chosen because most cases of undiagnosed diabetes in Greenland occur among adults [6,16]. We registered their age and gender. Individuals who were diagnosed with diabetes (coded with “D”) in their medical record before 1st of June 2010 were excluded from the study group. Among the remaining study sample we registered the number of persons screened with A1C and the number of persons who were actually diagnosed with diabetes during the 27 months period. The proportion of persons without diagnosed diabetes that was screened with A1C within the study period was used to describe the diagnostic activity.

#### Prevalence of diagnosed diabetes mellitus –study sample 2

The second study population was identified as all patients coded with “D” in the EMR using an electronically search module (data extraction September 2012). The age and gender specific prevalence was calculated using the Greenlandic population as of 1 January 2012 as the background population [21]. The prevalence of diagnosed diabetes mellitus among adults at or above 20 years old was estimated in the present study (2012)—as well as for 2008 and 2010 studies—based on data published in 2011 [17].

#### Statistics

Crude prevalence was reported for age and gender specific groups using 95% confidence intervals. Chi-square tests were used to compare frequencies between two groups, and chi-square tests for trends were used to examine prevalence rates with increasing age and over time. P-value at 0.05 was used as level of significance. The ethics committee for medical research in Greenland has approved the study with reference number 2013-5.

## Results

### Use of A1C as diagstic tool – study sample

**Figure 1:**
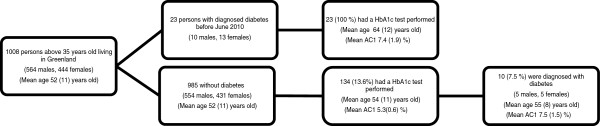
Flow chart showing the persons in the study sample 1.

A total of 1008 individuals born before the first of January 1978 on the first day of a month, living in Greenland as of September 2012 were identified (see Figure 1). Of those, 2.3% (23) were diagnosed with diabetes mellitus before the study period (before the first of June 2010). Among the remaining 985 individuals, 13.6% (134) were tested for diabetes mellitus utilizing measurement(s) of A1C at least once during the 27 months of observation. Diabetes mellitus was diagnosed in 7.5% (10) of the tested persons and in 1.0% of the whole group (985).

### Prevalence of diagnosed diabetes mellitus –study sample 2

**Figure 2:**
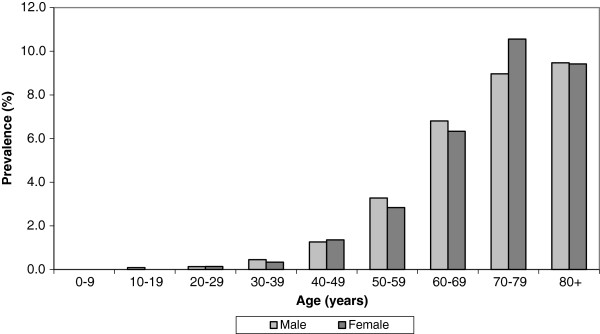
Age and gender specific prevalence of diabetes mellitus in Greenland 2012.

A total of 920 patients with diagnosed diabetes mellitus were identified. The age and gender specific prevalences of diabetes mellitus are illustrated in Table 1 and Figure 2. The total prevalence among adults aged 20–79 years old was 2.20% (95% CI: 2.05-2.34) with no significant difference between the genders (p=0.945). The prevalence increased with age for both males (p<0.001) and females (p<0.001). The prevalence among patients at or above 20 years old documented in this study is illustrated in Table 2 together with prevalence calculated from earlier published data [17]. The prevalence of diagnosed diabetes mellitus is still increasing in Greenland (p<0.001).

**Table 1:** Age and gender specific prevalence of diabetes mellitus in Greenland

**Age (years)**	**Male**	**Female**	**P**
	**Prevalence %**	**Prevalence %**	
	**(n/N) (95% CI)**	**(n/N) (95% CI)**	
**0-9**	0.0	0.0	
	(0/4205)	(0/3936)	-
	(0.0-0.0)	(0.0-0.0)	
**10-19**	0,1	0,0	
	(4/4322)	(0/4297)	0.046
	(0.0-0.2)	(0.0-0.0)	
**20-29**	0,1	0,1	
	(6/4530)	(6/4317)	0.933
	(0.0-0.2)	(0.0-0.3)	
**30-39**	0,5	0,3	
	(17/3757)	(11/3237)	0.457
	(0.2-0.7)	(0.1-0.5)	
**40-49**	1,3	1,4	
	(66/5210)	(60/4414)	0.691
	(1.0-1.6)	(1.0-1.7)	
**50-59**	3,3	2,8	
	(150/4575)	(101/3555)	0.258
	(2.8-3.8)	(2.3-3.4)	
**60-69**	6,8	9,3	
	(167/2452)	(111/1753)	0.538
	(5.8-7.8)	(5.2-7.5)	
**70-79**	9,0	10,8	
	(81/903)	(95/900)	0.257
	(7.1-10.8)	(8.5-12.6)	
**80+**	9,5	9,4	
	(16/169)	(29/308)	0.985
	(5.1-13.9)	(6.2-12.7)	
**20-79**	2,3	2,1	
	(487/21427)	(384/18176)	0.279
	(2.1-2.5)	(1.9-2.3)	

**Table 2:** Prevalence of diagnosed diabetes mellitus among adults at or above 20 years old in Greenland 2008-2012

**Year**	2008*	2010*	**2012**	**P**
Prevalence (%) among adult at or above 20 years old (number of patients/population)	1.41	1.85	2.29	<0.001
	(487/34,472)**	(721/39,008)	(916/40,080)	

*[19] **Ninety percent of the population included in 2008.

## Discussion

The principal finding in this study was that 13.6% of adults at or above 35 years have been tested for diabetes using A1C as the diagnostic tool, indicating that A1c is used as a diagstic tool in Greenland in this age group. Approximately, 7.5% of the tested persons were diagnosed with diabetes mellitus indicating that undiagnosed diabetes mellitus is still an issue and active case-finding searches relevant. Furthermore, the prevalence of diagnosed diabetes mellitus in Greenland documented in this study is the highest reported so far indicating increasing prevalence.

### Strength and weakness

The major strength in this study was that the whole population of Greenland was included. All A1C test results were to be found in the EMR. Case finding using whole blood capillary glucose was not included in this study. The actual number of patients tested for diabetes in the study period may therefore be underestimated. Furthermore, the increasing prevalence of diagnosed diabetes observed in the study period reflects the whole diagnostic activity in Greenland and not only the A1C diagnostic activity. Use of whole blood capillary testing may also have contributed to the diagnostic process because new guidelines can not be implemented immediately nationally.

Another limitation in this study is the unknown diagnostic activity of diabetes mellitus before introducing the A1C. Thus, the results mainly describe baseline diagnostic activity in Greenland after implementation of the new diagnostic criteria.

A possibly weakness in this study concerns the estimation of the prevalence of diagnosed diabetes mellitus. Only patients that were actually coded with ”D” in the electronic medical record were included in the study. This may lead to an underestimation of the true prevalence of diagnosed diabetes mellitus. However, patients without a ”D” code who receive prescriptions electronically for glucose lowering drugs, have their medical record reviewed annually, a process intended to identify patients with diabetes yet no “D” code. Thus the underestimation is not likely to be of any significant magnitude.

### Use of A1C as diagstic tool

The use of A1C as diagnostic toolwithin the first 27 month after introducing the new strategy underlines the feasibility in a Greenlandic context of using this method. A1C testing can be performed in contrast to fasting whole blood glucose tests or oral glucose tolerance tests without the delay of waiting on fasting individuals. On the other hand it is obvious that the diagnoses using A1C criteria are not exactly the same as those diagnosed on plasma glucose [19,23-25]. Also, ethnical differences have been suggested as proving problematic when using HbA1c diagnosis. Thus, in most cases a lower number of patients are diagnosed using A1C criteria than when using oral glucose tolerance tests. This discrepancy has also been demonstrated among Greenlanders where the prevalence of diabetes based on oral glucose tolerance test resulted in 7.0% compared to 3.9% using the A1C criteria [24]. Similary, the prevalence of diabetes based on oral glucose tolerance compared to A1C criteria was higher in United Kingdom (3.7% versus 1.0%), Australia (4.0% versus 0.7%) and Kenya (3.4% versus 1.4%) while the opposite was the case in Denmark (4.2% versus 6.7%) and India (10.2 5 versus 12.9%) [24].

However, the obvious convenience of A1C might increase the number of patients tested and diagnosed with diabetes tested [23]. Furthermore, it seems evident that the most severe cases and consequently those that might benefit most from treatment are those that are diagnosed using A1C [26-29]. Using A1C as a diagnostic tool instead of the former use of whole blood capillary glucose (and not plasma glucose as recommended internationally) brings the diagnostic procedure in accordance with the most recent international guidelines [18].

Finally, in a sparsely populated country like Greenland with small health care units with a fluctuating health care staff, it is imperative to have a very feasibly and reliable diagnostic setup for an upcoming disease like diabetes.

### Prevalence of diagnosed diabetes mellitus

Increasing prevalence of diabetes is seen all over the world. In accordance with that we have found an increasing prevalence in this study compared to previous studies [17].

The increase has been documented withih the last five years and is most likely due to increased diagnostic activity. Improved treatment and increased survival among patients with diabetes along with an actual increase in the prevalence can also contribute to an increasing overall prevalence.

However, in a global perspective the prevalence of diagnosed diabetes remains quite low [30]. Undiagnosed patients still seem to be quite common. Perhaps therefore the 7.5% of individuals tested for DM were actually diagnosed with DM, which could indicate that the overall percentage of undiagnosed patients with DM was around 7.5% - around the double of the prevalence of diagnosed DM However, most likely, the individuals tested with A1C are not a random sample of the population and rather represent individuals with increased risk of diabetes, making the percentage of undiagnosed cases among the total population less than 7.5%.

## Conclusion

In conclusion, testing for DM using A1C as diagnostic tool is used in Greenland. The prevalence of diagnosed DM in Greenland although increasing remains low in a global perspective. Undiagnosed DM may still be an important issue in Greenland.

## Abbreviations

DM: Diabetes mellitus; A1C: Haemoblobin A1C; CI: Confidence intervals; EMR: Electronic medical record.

## Competing interests

The authors have no competing interest to declare.

## Authors’ contributions

The study was designed by MLP. Both LD and MLP participated equeally in the analysis of data. First draft of the manuscript was carried out by LD and rewritten by MLP. Both authors read and approved the final manuscript.
